# FSH *in vitro* versus LH *in vivo*: similar genomic effects on the cumulus

**DOI:** 10.1186/1757-2215-6-68

**Published:** 2013-09-25

**Authors:** Mourad Assidi, François J Richard, Marc-André Sirard

**Affiliations:** 1 Département des Sciences Animales, Faculté de l’Agriculture et de l’Alimentation, Université Laval, Québec, QC G1K 7P4, Canada; 2 Center of Excellence in Genomic Medicine Research, King AbdulAziz University, Jeddah 21589, KSA

**Keywords:** Transcriptomic overlap, Genomic substitution, Gonadotropin molecular signalling

## Abstract

The use of gonadotropins to trigger oocyte maturation both *in vivo* and *in vitro* has provided precious and powerful knowledge that has significantly increased our understanding of the ovarian function. Moreover, the efficacy of most assisted reproductive technologies (ART) used in both humans and livestock species relies on gonadotropin input, mainly FSH and LH. Despite the significant progress achieved and the huge impact of gonadotropins, the exact molecular pathways of the two pituitary hormones, FSH and LH, still remain poorly understood. Moreover, these pathways may not be the same when moving from the *in vivo* to the *in vitro* context. This misunderstanding of the intricate synergy between these two hormones leads to a lack of consensus about their use mainly *in vitro* or in ovulation induction schedules *in vivo*. In order to optimize their use, additional work is thus required with a special focus on comparing the *in vitro* versus the *in vivo* effects. In this context, this overview will briefly summarize the downstream gene expression pathways induced by both FSH *in vitro* and LH *in vivo* in the cumulus compartment. Based on recent microarray comparative analysis, we are reporting that *in vitro* FSH stimulation on cumulus cells appears to achieve at least part of the gene expression activity after *in vivo* LH stimulation. We are then proposing that the in vitro FSH-response of cumulus cells have similitudes with the in vivo LH-response.

## Introduction

Mammalian female reproductive function is finely regulated by a set of coordinated endocrine signals that allow successful events of oocyte developmental competence, granulosa cells differentiation, extracellular matrix (ECM) production, ovulation, fertilization, and early embryonic development. Gonadotropins (mainly FSH and LH) are the main extra-ovarian endocrine factors involved in the control of these ovarian functions [1-6]. The release of these two anterior-pituitary hormones is governed by the hypothalamus via the GnRH (gonadotropin-releasing hormone) and modulated by other ovarian factors such as activin and inhibin [7]. Following a gonadotropin-independent phase, the mammalian follicular growth first becomes FSH-dependent at the secondary stage and then LH-dependent prior to ovulation [8-12]. While FSH is mainly involved in follicular growth, cellular proliferation and oestrogen production (aromatase activity), LH induces androgen biosynthesis, final maturation of the oocyte and ovulation [13-19]. To achieve their functions, FSH and LH trigger multiple downstream cascades of intra-ovarian pathways that are necessary for proper female fertility [13,14,17,20-23]. In addition, it has been shown that the efficiency of most assisted reproductive technologies (ART) used in both humans and livestock, including ovarian stimulation and oocyte *in vitro* maturation (IVM), relies on gonadotropin input [24-31].

Interestingly, successful gonadotropin-induced maturation of oocyte was shown to require *de novo* mRNA synthesis in follicular somatic cells. This gene expression activity aims at supplying the oocyte and follicular cells with crucial factors to achieve subsequent events of maturation and ovulation [32-35].

*In vitro*, it has been demonstrated that FSH improves oocyte maturation (both nuclear and cytoplasmic), cumulus cell (CCs) expansion, *in vitro* fertilization (IVF) and early embryo development in several mammalian species including cattle [30,36,37], mouse [38], pig [39-41] and human [22,42,43]. Since FSH has improved significantly the oocyte maturation and developmental competence, its receptor, FSHR, was assumed to be expressed in mural granulosa cells (MGC) and CCs starting at the secondary follicular stage in most mammals, including mice, pigs, sheep, cows and humans [13,40,44-47]. FSH is also used to trigger the follicular growth during the preovulatory stage. Once they reach the fully grown stage, the superovulation is thereafter induced by LH in livestock animals and human [25,27,28,48-50]. Assuming that functional LH receptors are absent in CCs in the *in vitro* context [37,51,52], FSH has been the major gonadotropin used in IVM to trigger oocyte maturation fulfillment [16,30,53].

To be effective, LH signaling thus depends on the expression of functional luteinizing hormone/choriogonadotropin receptor (LHCGR) in the follicle. LHCGR expression was reported in theca and granulosa cells [54-56] but was absent in both oocytes and CCs [37,51,52]. Therefore, the meiotic induction effect of LH on CCs was recently proposed to be indirectly mediated through the EGF-like factors [18,20,56,57]. The addition of LH into the IVM media might therefore not be needed in vitro [30,47,58]. However, Peng et al. [59] have reported the expression of LHCGR in rat CCs after PMSG stimulation. Similar findings confirmed this LHCGR expression in cumulus cells downstream the FSH pathway in human [60] and pig [31,61] both in vivo and vitro, raising therefore a controversy that needs further exploration.

In this review, we attempt to briefly address the general pathways of FSH and LH in follicular cells (mainly in CCs) that result in downstream transcriptional activity. Special focus will be given to the common features between the transcriptionally upregulated genes through FSH *in vitro* versus their LH counterparts *in vivo*. Based on common structural and functional features between FSH and LH, the hypothesis of partial replacement or “compensation” of LH action by FSH *in vitro* is explored. Using several findings reported in previous studies and microarray data in our laboratory, we propose herein an interesting aspect of the gonadotropin actions that may increase our understanding of their molecular pathways as well as their intricate synergy.

### Gonadotropin-mediated gene expression and oocyte developmental competence

In view of the gonadotropins’ beneficial effects, they are used both *in vitro* and *in vivo* to improve oocyte developmental competence. Although their molecular mechanism of action remains ill-defined, we supposed that their genomic effects *in vitro* could be different from those *in vivo*, where they act in synergy and where both granulosa and theca cells are present. This hypothesis emerged from the difference in blastocyst rates between *in vivo* and *in vitro* oocyte maturation. For example, in cows, if the follicular development is supported by FSH, the rate of oocytes with successful developmental competence *in vivo* is between 60 and 80% [27,28]. In contrast, if oocytes are recovered from unstimulated antral follicles (slaughterhouse), this percentage drops to an average of 25 or 45% in ideal IVM conditions (IVM Schedule: 6h with FSH + 16 to 18h without any hormone supplement) [30,62]. It is clear that the *in vivo* context, which includes the sequential effects of gonadotropins (FSH & LH) and the presence of other intrafollicular factors, is far more suitable to oocyte competence acquisition. Interestingly, data in our laboratory and elsewhere showed that adding FSH to IVM media allowed an increase in blastocyst rate equivalent to half of the in vivo maturation success rates [45,63]. To explain this increased development in absence of an LH surge but in presence of FSH (using recombinant human FSH (rhFSH) without contamination risk), we assume that FSH is able to accomplish its own biological function and to substitute at least part of the effects of LH, resulting in developmental competence of some oocytes. In the absence of LH, FSH appears to be able to exert key functions normally achieved by LH. Our preliminary data comparing the transcriptomic effects of FSH *in vitro* versus LH *in vivo* highlight the necessity of further investigation to demystify the molecular overlap between FSH and LH. Concerning the cell signaling and although, the high doses of FSH in vitro will increase significantly the cAMP in mammalian cumulus cells and downstream pathways [64-67] (which may mimic the LH preovulatory effect), this second messenger rise is not enough to explain this important FSH functional substitution of the LH effect.

This possible compensation/substitution of the *in vivo* effects of LH by FSH *in vitro* could be explored at many levels (metabolic, physiological, morphological, transcriptomic, etc.). The present work is an overview of possible transcriptomic compensation of LH by FSH *in vitro* and briefly reviews their respective signaling pathways that may induce gene expression, followed by a case report of genomic effect comparison of FSH *in vitro* versus LH *in vivo* in bovine CCs.

#### Main signaling pathways of FSH in vitro

It has been shown that FSH is a key regulator of ovarian function, in particular follicular growth and granulosa cell differentiation [11,68]. FSHβ-deficient mice were unable to develop past the preantral stage [11]. These observations confirm that folliculogenesis is gonadotropin-dependent starting at the antral stage. The main functions triggered by FSH in the mammalian ovary are cell proliferation, apoptosis prevention, estradiol production, cell secretion, and regulation of several other genes [5,16,38,61,69-71]. Additionally, high doses of FSH are an essential ingredient in IVM media and was shown to efficiently promote full oocyte maturation in several mammalian species *in vitro* including cow [30,33], mouse [72] and pig [4,73]. This *in vitro* FSH effect is initiated in cumulus-oocyte complex (COCs) via its receptor (FSHR) on cumulus cells (CCs). It has been known for decades that FSH has no effect on denuded oocytes in vitro. FSHR is a GPCR (G-protein-coupled receptor) with a specific seven-transmembrane domain that was shown to activate the classical FSHR/AC/cAMP/PKA pathway. Among the two activated isoforms of PKA, only PKAII was shown to be involved in the transcriptional events in CCs required for meiosis resumption (GVBD) [74,75]. This *de novo* gene expression is indispensable for gonadotropin-induced oocyte maturation in murine and feline species [75,76] and was shown to involve the MAPK downstream of the cAMP-dependent PKA pathway in most mammals including mouse [77,78], rat [79] and cow [80]. In fact, it was shown that PKA phosphorylation and, EGF-like factors overexpression and secretion are both required to the FSH activation of ERK1/2 in pig and mice cumulus cells [61,81-83]. Additional signaling cross-talk events between the cAMP/PKA and the EGFR-MAPKs cascades were shown to up-regulate the PKA pathway via the synthesis of PGE2 [84-86]. Additionally, the inhibition of the MAPK pathway in CCs (or COCs) impaired gonadotropin-induced oocyte maturation and prevented the over-expression of crucial genes, such as PTSG2 and HAS2, required for oocyte maturation fulfillment, CC expansion, and steroidogenesis [87-89]. Interestingly, this MAPK effect is dependent on the PKA pathway but also on some oocyte paracrine factors reported to induce the EGF-like factors in CCs [90]. Additional PKA gene expression activity was associated with its two catalytic subunits that were able to transit to the CC nucleus. Several key genes were reported to be expressed downstream of this pathway, including HAS2, TNFAIP6, PTGS2, CYP19A1 and EGF-like factors ([75,91-96], recently reviewed in [20,21]). This transcriptional activity was mediated mainly – but not exclusively – through phosphorylation of CREB (CRE-binding protein) and therefore its binding to the CRE (cAMP-responsive-element) region in the promoter [97]. Additional transcription factors including AP1, SP1 and C/EBP family were also reported to contribute in the transcriptomics action of FSH [16,98,99]. FSH-induced PGE2 secretion is also an additional indirect effect that maintains the cAMP levels and stimulates the overexpression of the EGF-like factors [81].

FSH-mediated gene expression activity also occurs in a PKA-independent manner. In fact, it was demonstrated that FSH phosphorylates PKB/Akt and SGK1 via the PI3K (phosphatidylinositol-dependent kinase)/PDK1 (phosphoinositide-dependent kinase1) pathway in rat granulosa cells [100], mouse CCs [101,102] and porcine CCs [103], to support oocyte maturation *in vitro*. Interestingly, the PI3K/PKB pathway downstream of FSH was shown to induce cell survival and progesterone production in porcine CCs [31,103].

PKC was also reported to mediate the effects of FSH in CCs by activation of MAPK. This PKC action upstream of the MAPK pathway (and possibly through other pathways) induced the expression of key factors (*de novo* proteins) required for meiotic maturation of the oocyte, including the EGF-like factors in most mammalian species [45,104-106]. Similar effects induced by PMA (phorbol 12-myristate13-acetate), which is a PKC activator, were shown in CCs. Moreover, induction of the PKC pathway by FSH was associated with the mobilization of intracellular calcium that is assumed to be favorable to oocyte maturation and subsequent fertilization [23,104,107-110].

*In vivo,* the EGF like factors overexpression in CCs occurred following the action of LH-induced PGE2 secreted from granulosa cells through the PKA/CREB/MAPK pathway. This aforementioned pathway raised PGE2 production also in CCs which in turn amplify the local expression of EGF like factors amplification [18,20,56,57]. In vitro, EGF like factors and in presence of FSH (or forskolin) *in vitro* were reported to play a crucial role in IVM. Taken together, the EGF like factors are key players involved in gonadotropin-induced maturation in mammalian COCs [81,82,105,111,112] and probably further fertilization [113]. In fact, the EGF-like factors were shown to act in a positive regulation loop to overexpress PGE2 in porcine cumulus cells [81]. The PGE2 will thereafter by autocrine effect increase the EGF-like peptides that triggered gene expression in CCs through the extracellular signal-regulated kinases (ERK1/2/3) and PGE2 pathways [89,91,114,115].

FSH was also able to rapidly (within 1 hour) activate the MEK/MAPK pathway in mouse CCs to allow oocyte maturation [77]. The most studied MAPK are ERK1/2, JNK/SAPK *(c-jun*terminal kinase/stress-activated protein kinases) and p38MAPK. Several transcription factors were reported to act downstream of the MAPK and ERK including AP1 and ATF2, CMYC [115,116]. In this context, P38MAPK was also phosphorylated by FSH through the cAMP/Epac(exchange protein activated by cAMP)/Rap (Ras-like related proteins)/Raf pathway, which is PKA-independent [100]. ERK1/2 was also involved in mural granulosa cells and CC steroidogenesis (progesterone and estradiol) induced by FSH [117,118]. Once produced, these steroids, mainly progesterone, were shown to promote gene expression and contribute to oocyte competence and CC expansion [119-121].

Several studies were performed to assess the gene expression patterns in follicular cells induced by FSH *in vitro*. These sets of genes induce numerous biological and molecular functions associated with cell signaling, CC expansion, steroidogenesis, gene expression, etc. [23,34,61,91,98,122-125]. Analysis of these gene expression patterns has yielded insights into the molecular involvement of FSH in CC function leading to oocyte developmental competence acquisition.

#### Do cumulus cells express LHCGR?

Before reviewing the LH pathways, in particular those leading to gene expression in CCs, it is important to discuss available data about the possible expression of LHCGR in this compartment. Some studies have reported the absence of LHCGR in CCs [37,51,52]. The addition of LH in FSH-based media for COC maturation *in vitro* therefore does not improve oocyte developmental competence [30,37,126]. In contrast, other studies documented LHCGR expression in CCs, suggesting that LH might have a direct effect [59,127]. Additional evidence seem to confirm these aforementioned findings in CCs of several mammalian species including pig [31,61], mouse [128,129], rat [130], cow (isoform E) [55] and human [131]. Beneficial effects of LH on *in vitro* embryo yields were even shown but the amount of LH used (1ug per ml) was likely contaminated by enough FSH (1%) to questions the conclusion [132,133]. These opposite findings may be due to differences in several parameters such as the COCs’ follicular stage, the tissue type (granulosa or cumulus), the gonadotropins’ origin (recombinant versus purified) and the detection technique and its sensitivity. To resolve this issue, the analysis of the protein functionality is required as several isoforms of the LH receptors are present in granulosa and cumulus cells [55,60,134,135]. It is also possible that the appearance of such receptor the cumulus is follicle size dependant or follicle differentiation dependant [60,134] creating an ambiguous response when pools are used. In the same way, the expression variation of particular LHCGR isoforms in CCs according to the follicular stage could also be the cause of this discrepancy. The analysis of a limited isoform’s population may be insufficient to confirm the absence of these receptors in CCs. In the mouse, the oocyte is believed to control the mRNA stability for the LH receptor. Sufficient data about the differential expression of LHCGR according to both the cell subtype (theca, granulosa or cumulus) and the follicular stage is still lacking. Possible reconciliation that reinforces our hypothesis was reported recently by the sequential culture system (FSH followed by LH) suggested by Kawashima *et al*. [61]. In this study and elsewhere, FSH was shown to trigger the expression of functional LHCGR that could respond to the subsequent action of LH and result in greater developmental competence until the blastocyst stage both *in vivo* and in vitro culture [129].

#### Main gene pathways induced by LH in vivo

Similarly to FSH, the contribution of LH in follicle dominant selection, oocyte final maturation, ovulation and subsequent fertilization was studied. In fact, LH is necessary in the selection of the dominant follicle in cattle and horse ([136] for review). This dominance is marked in cattle by an increasing dependence of the follicle on LH, mainly at the signalling and transcription levels [137,138]. While only FSH was able to induce CC expansion *in vitro*, LH and hCG were able to promote this mucification *in vivo* through the Ras/Raf/MAPK pathway (downstream of cAMP) as well as oocyte maturation [1,31,78,139,140]. Dr. Richards’ group has recently shown *in vitro* that the LH-induced transcriptional events are required for oocyte maturation, CC mucification, ovulation and luteinization are induced through activation of some downstream transcription effectors such as C/EBPβ (CCAAT/Enhancer-binding protein-beta) via the ERK1/2 pathway [115], reviewed in [21,125,141,142].

Like FSH *in vitro*, LH was also shown to activate the PKAII isoform which triggers gene expression events required for oocyte maturation fulfillment [143-145]. Additionally, LH mediates the overexpression of the EGF-like factors mainly EREG, AREG (through the p38 MAPK), BTC and NRG1 (via the C/EBP). These growth factors propagate and amplify the LH signal in CCs as previously suggested ([56,89], reviewed in [114,146]). Other key genes were also induced by LH, notably those involved in CC expansion and prostaglandin synthesis such as HAS2, TNFAIP6, PTX3, CSPG2, PTSG2, etc. [147]. Knockout of these crucial genes in mouse causes severe defects in the animal reproductive phenotype and subsequent fertility (reviewed in [21].

In porcine and bovine CCs, LH was also shown to induce steroidogenesis, mainly progesterone and estradiol [15,148]. LH receptor null mice were infertile with defective steroid production [149,150]. Moreover, the gene expression patterns in CCs were deeply affected in PGR null mice supporting a key transcriptional role of progesterone in oocyte maturation and subsequent ovulation and fertilization. Moreover, PGR is a nuclear receptor that acts as a transcription factor to mediate the LH ovulatory response by the expression of key genes such as ADAMTS1 and Catepsin L [151] for reviews [87,125,152-154]. The inhibition of PGR or progesterone action prevents meiosis resumption and ovulation in pig [155,156]. Moreover the PRKO mice are unable to spontaneously ovulate, but when the oocytes are punctured and collected prior to ovulation, they are able to reach the blastocyst stage [154,157,158].

In addition to the PGR, LH surge also induces various other transcription factors leading to diverse transcriptional effects and physiological responses [152]. The PKC pathway was suggested as a possible transduction mode of this LH stimulation [159]. PKC epsilon was furthermore shown to induce a survival (anti-apoptotic) effect on human CCs downstream of the PI3K/Akt pathway [160]. This PKC action is possibly associated to the reported LH-induced intracellular rise in calcium in follicular cells [161,162].

Concerning oocyte competence, the presence of LH receptor is associated with the dominant status in bovine and other mono-ovulating species [9,136,163]. The dominant follicle in bovine occurs at the average diameter size of 8.5 mm and is associated with an increase capacity to generate competent oocyte. In contrast, the follicles used for this study and in almost all study using in vitro maturation of bovine oocytes are much smaller, normally from 3-5mm size. In these follicles the cumulus has not been in contact with granulosa cells responding to any LH signalling although theca cells do have LH receptors at the 3mm size stage [164,165]. The oocytes from such small tertiary follicles have a limited capacity to reach the blastocyst stage in vitro after IVM-IVF and culture. The addition of FSH in vitro at low doses in a two step IVM culture system (6 h with rhFSH (0.1 μg/ml) + 18 h without hormones) in order to mimic the preovulatory intra-follicle conditions before the LH surge allowed an increase of the oocyte competence from around 20% to close to 45% [45].

Therefore the FSH effect in vitro on cumulus cells looks to accomplish its role and mimics the *in vivo* LH-ovulatory effect especially at higher doses. The rise in developmental rate in vitro is similar between oocytes from follicles with or without LH [30]. No report has been published yet on the comparison of cumulus transcriptome from dominant follicles compared to smaller ones as used in our study.

Human chorionic gonadotropin (hCG) is another gonadotropin that has high affinity for the LH receptor, named for this reason the LHCGR. In addition to the same α-subunit shared between LH and hCG, this affinity is primarily due to high similarities between the two β-subunits. Interestingly, hCG is able to trigger most of the LH effects for longer periods due to its greater half-life [166]. This property is desired in the ovarian stimulation drugs since it allows more time, flexibility and management possibilities during the ovarian stimulation programs, particularly in human IVF. For these reasons, hCG has often been used instead of LH due to its LH-like effect (reviewed in [167]).

Analogous to the FSH effect, the LH activation of several signal transduction pathways in CCs leads to diverse but well organized *in vivo* transcriptional responses that contribute to suitable oocyte competence acquisition, subsequent ovulation and fertilization and early embryo development. These beneficial effects were confirmed both *in vivo* and *in vitro*[18,28,56,168] and reviewed by [17,26].

#### Comparative analysis of FSH and LH pathways

Despite their specific biological functions, FSH and LH share some interesting similarities. In fact, both are pituitary-derived glycoproteins composed of a heterodimer of two subunits (α and β). These two subunits are linked by non-covalent bonds. While the α-subunit is common between all pituitary gonadotrophins, the β-subunit is specific, and, importantly, binds to the receptor, thus it exerts the biological effects [14]. Expression of these subunits is differentially induced by the pulsatile gonadotropin-releasing hormone (GnRH) via the PKC/MAPK signaling pathway [169]. Interestingly, both FSH and LH exert their stimulatory effects through a seven transmembrane receptor (7TMR). These receptors are members of the G protein-coupled receptors (GPCR) family that stimulate several signaling pathways mainly through G proteins [170-172]. Moreover and as discussed before, the two gonadotropins are able to induce gene expression events by targeting numerous transcription factors downstream of key signaling pathways, such as PKA, PKC, PKB/Akt, MAPK and PI3K. These transcriptional activities of FSH *in vitro* or LH *in vivo* are essential to achieve successful oocyte developmental competence. These similarities in molecular structure, the specific receptor, and the transcriptional pathways and gene targets lend support to the hypothesis of possible overlapping genomic roles between these two gonadotropins.

### Evidence of genomic effects’ similitude between LH in vivo and FSH *in vitro*

In addition to their structural and functional (receptor and downstream pathways) similarities, the goal here is to look for common genes that are transcriptionally upregulated (directly or indirectly) by the two gonado-tropins and that could support our hypothesis of an LH-like effect of FSH in vitro. These transcriptomic similitudes between the action of LH in vivo and FSH *in vitro*. To this end, we compared the FSH-induced genes in CCs in conditions associated with an increase of oocyte competence *in vitro*[23] to the *in vivo* context 6 hours after the LH surge. Because finding a timeline to compare cumulus cell status and gene expression patterns *in vivo* versus *in vitro* can be difficult, we used the meiotic status of the oocyte as a suitable reference. Thus, our analysis focused on the comparison of CCs gene expression patterns *in vitro* at 6 hours of IVM (oocyte entering GVBD stage) [23] versus the overexpressed genes in CCs at 6 hours after the LH surge *in vivo* (when the oocyte is again entering the GVBD stage) [173].

**Figure 1:**
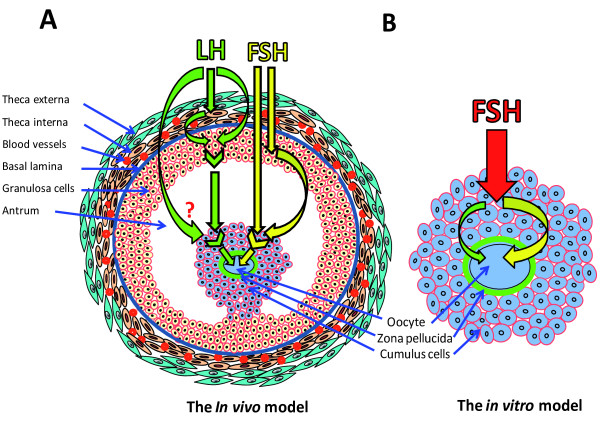
**Model of gonadotropin-mediated induction of oocyte competence in vivo versus in vitro: In addition to its own effects, could FSH in vitro substitute for some of the effects of LH?*** FSH: follicle stimulating hormone; LH: luteinizing hormone.*

**Figure 2:**
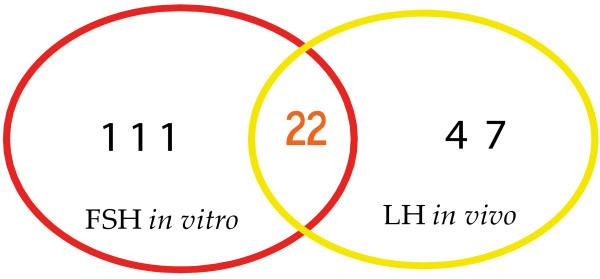
**Common genes overexpressed by FSH in vitro versus LH in vivo in bovine CCs around the GVBD, as revealed by microarray.***FSH: follicle stimulating hormone; LH: luteinizing hormone.*

For the *in vitro* study, the focus was on the definition of the FSH-induced gene expression effect in bovine CCs (around GVBD) associated with oocyte competence *in vitro*[23]. Although the whole molecular pathway of FSH action in CCs is not fully defined, we supposed that this genomic effect in *vitro* (Figure 1B) could be different from its counterpart *in vivo*, where FSH acts in synergy with LH (Figure 1A). The *in vitro* versus *in vivo* differences in blastocyst outcome support this assumption. Concerning the subsequent *in vivo* study, we have analyzed the LH-induced gene expression effect *in vivo* (close to the GVBD) again on bovine CCs [174]. This latter *in vivo* context should better reflect the real mechanism of CC contribution to oocyte competence acquisition. In fact, LH was reported to induce final maturation of the oocyte *in vivo* by acting on CCs which express and deliver competence inducers to the oocyte [28,59]. Keeping all these considerations in mind, our analysis focused on the comparison of the genomic action of FSH *in vitro* versus LH *in vivo*. A non-exhaustive list of common molecular genes between LH and FSH, expressed in CCs, and associated with oocyte final maturation is provided (Table 1). Among the 133 significant candidates induced by FSH *in vitro*, 22 genes were also induced by LH *in vivo*. This means that *in vivo*, LH is able to induce the transcription of around 16.5% (22/133) of all the genes overexpressed by FSH *in vitro*. Strikingly, these common candidates correspond to almost 32% (22/69) of the genes overexpressed via LH *in vivo* (Figure 2).

**Table 1:** Common overexpressed genes in bovine CCs around the GVBD between FSH in vitro versus LH in vivo

**No.**	**Gene name**	***Gene/protein full name (if available)***	**Accession no.**
1	ATP1B4	Bos taurus ATPase, (Na+)/K + transporting, beta 4 polypeptide	NM_001101919
2	ATP6V1C1	Bos taurus ATPase, H + transporting, lysosomal 42kDa, V1 subunit C1	NM_176676
3	BAMBI	Bos taurus BMP and activin membrane-bound inhibitor homolog	NM_001046309
4	HSPA8	Bos taurus heat shock 70 kDa protein 8	NM_174345
5	INHBA^*^	Bos taurus inhibin, beta A	NM_174363
6	PAPD1	Bos taurus PAP associated domain containing 1	BC104501
7	PSMA2	Bos taurus proteasome (prosome, macropain) subunit, alpha type, 2	BC102206
8	RHOA	Bos taurus ras homolog gene family, member A	NM_176645
9	RPL3	Bos taurus ribosomal protein L3	BT021012
10	SELK	Bos taurus similar to selenoprotein K	BC108150
11	SLC25A5	Bos taurus solute carrier family 25 member 5	BC102950
12	TNFAIP6^*^	Bos taurus tumor necrosis factor, alpha-induced protein 6	NM_001007813
13	UBA6	Bos taurus ubiquitin-like modifier activating enzyme 6	NM_001083438
14	CHSY1	Homo sapiens carbohydrate (chondroitin) synthase 1	NM_014918
15	EREG^**●**^	Homo sapiens epiregulin	NM_001432
16	FOXO3A	Homo sapiens forkhead box O3 (FOXO3), transcript variant 2, mRNA	NM_201559
17	PGR^*^	Bos taurus progesterone receptor	NC_007313.4
18	NEAT1	Homo sapiens nuclear enriched abundant transcript 1	EF177379
19	SGMS2	Homo sapiens sphingomyelin synthase 2 (SGMS2), transcript variant	NM_001136258
20	AGPAT9	PREDICTED: Bos taurus similar to 1-acyl-sn-glycerol-3-phosphate O-acyltransferase 9	XM_597964
21	RBMX	PREDICTED: Bos taurus similar to Heterogeneous nuclear ribonucleoprotein G	XM_875611
22	SLC39A8	PREDICTED: Bos taurus similar to Solute carrier family 39 (zinc transporter), member 8	XM_584935

Real-time PCR validation of overexpression following FSH *in vitro*(*) or LH *in vivo* (^**●**^); α = 0.05%.

These findings mean that about one third of the genes induced by LH *in vivo* could be induced by FSH *in vitro*. FSH action *in vitro* therefore seems to act for both its own and LH’s *in vivo* functions [16]. Using its common downstream pathways of gene expression with LH, FSH *in vitro* appears to reproduce its in vivo function and substitute at least partially for the in vivo activity of LH (Figure 1B).

**Figure 3:**
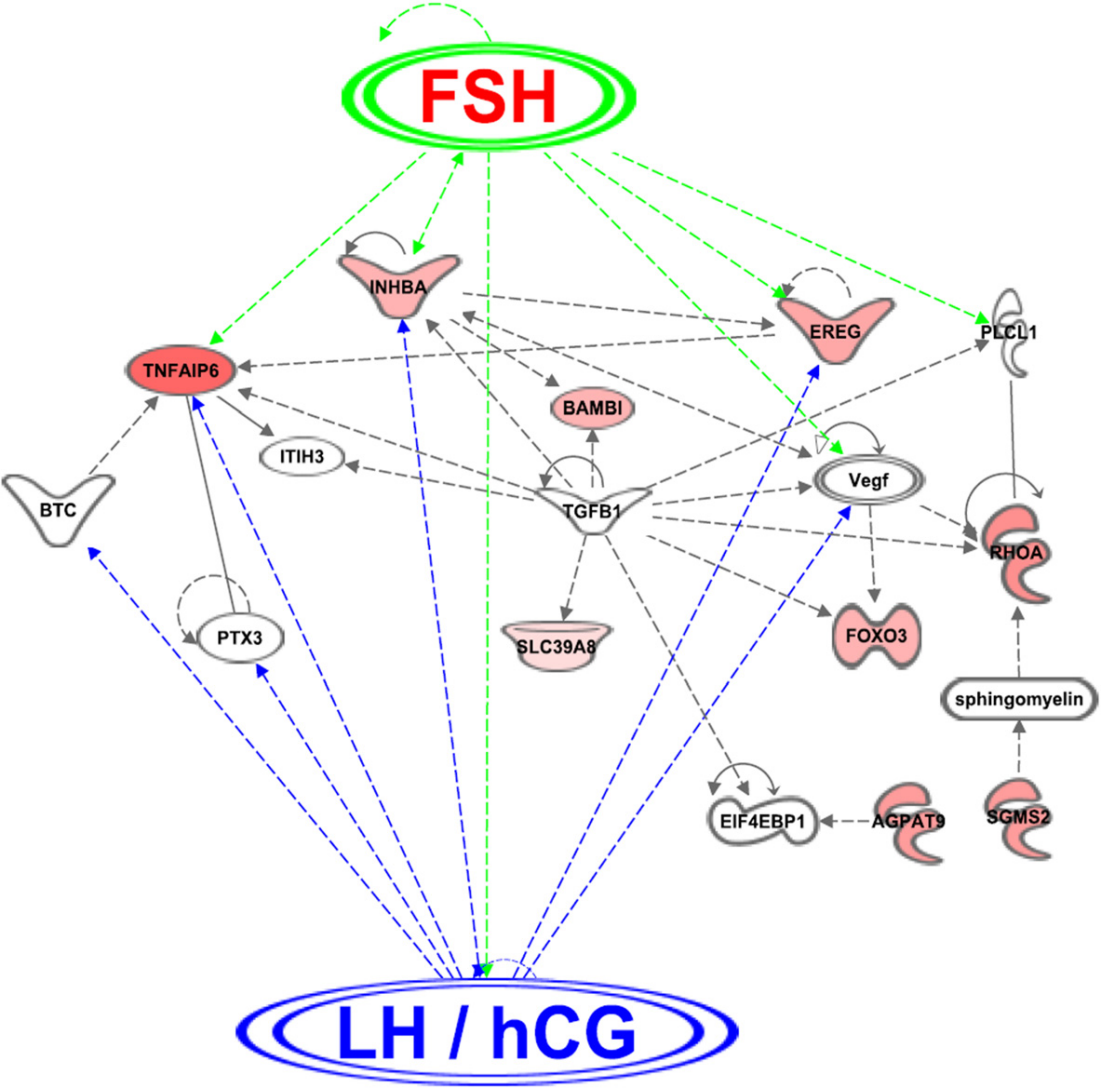
**A summary of a gene network including common gene candidates induced by both FSH in vitro and LH/hCG in vivo as revealed by the IPA software.*** FSH: follicle-stimulating hormone; LH/hCG: luteinizing hormone/human chorionic gonadotropin; EREG: epiregulin; TNFAIP6: tumor necrosis factor, alpha-induced protein 6; INHBA: inhibin, beta A; BAMBI: BMP and activin membrane-bound inhibitor homolog (Xenopus laevis); FOXO3: forkhead box O3; RHOA: ras homolog gene family, member A; TGFB1: transforming growth factor, beta 1; PTX3: pentraxin-related gene, rapidly induced by IL-1 beta; BTC: betacellulin; ITIH3: inter-alpha (globulin) inhibitor H3; SLC39A8: solute carrier family 39 (zinc transporter), member 8; VEGF family: vascular endothelial growth factor; PLCL1: phospholipase C-like 1; SGMS2: sphingomyelin synthase 2; AGPAT9: 1-acylglycerol-3-phosphate O-acyltransferase 9; EIF4EBP1: eukaryotic translation initiation factor 4E binding protein 1.*

Analysis of the gene networks of the 22 common genes, using the gene Ingenuity Pathway Analysis (IPA) software (Ingenuity Systems, http://www.ingenuity.com/products/ipa; [175], confirms the high overlap between FSH and LH at the transcriptional level. Figure 3 illustrates the gene network with highest score following the IPA analysis. In this network, several key gene pathways involved in oocyte competence, steroidogenesis, CC differentiation and mucification, ovulation and luteinization were activated by both FSH and LH. Surprisingly, most of these common target genes (and therefore their functions) are influenced by the TGFbeta factors (Figure 3). These growth factors in the follicular context may correspond to the oocyte-secreted factors (mainly GDF9 and BMP15) reported as crucial factors in the oocyte-follicular cells dialog [176-178].

The progesterone receptor (PGR) is another interesting candidate commonly expressed in response to FSH and LH (Table 1). These findings are in line with recent reports in bovine cumulus *in vitro* confirming gonadotropin induction of PGR expression [23,151,179]. This receptor is also essential in reproduction and particularly in the ovulatory process through stimulation of the expression of enzymes crucial to ovulation such as ADAMTS1 and CTSL (cathepsin L), and the inhibition of follicular cell apoptosis [179,180]. Moreover, PGR is involved in intracellular signaling and kinase activities required for oocyte maturation and subsequent ovulation [87,121,181]. This nuclear receptor has a transcriptional role in mediating gonadotropin stimulation by downstream expression of several key genes. These observations were confirmed by important alterations of CC gene expression patterns and crucial signaling pathways in PGR null mice [89,153,158,182].

To our knowledge, this is the first time that the mimicking of LH action in vivo by FSH *in vitro* is highlighted at the transcriptomic level. It is important to note that this analysis was made using a custom-made microarray [23]. It is expected that the number of common candidates between the two gonadotropins may increase if commercial whole genome microarrays were used.

### Concluding remarks

These similarities between FSH effect in vitro and LH in vivo, although still preliminary, support our hypothesis of potential functional substitution between FSH and LH. They are also consistent with previous results where the addition of LH to FSH-based IVM media did not result in any additive effect either in cumulus expansion or in oocyte competence as measured by the blastocyst yield. Moreover, this probable functional mimetic action of LH function by FSH *in vitro*, should help in improving *in vitro* culture systems and ovulation induction programs through a better understanding of the FSH/LH synergy *in vivo*. Furthermore, these common candidates will serve as a precious preliminary tool to monitor such mimetic action and should advance our understanding of the molecular pathways that lead to successful oocyte maturation, cumulus cells differentiation, ovulation and subsequent fertilization.

However, additional studies are required to confirm our results including the overexpressed and underexpressed genes, and to investigate the FSH/LH synergy. Studying the gene expression patterns induced by FSH, LH and (FSH + LH) in sequential culture system could be an interesting way to validate these findings.

## Competing interests

The authors declare that they have no competing interests.

## Authors’ contributions

All this study was achieved at Laval University. MAS designed the study. MA contributed in the study design, analyzed the data and drafted the manuscript. Both MAS and FJR were involved in data interpretation, performed a critical revision of the manuscript and gave their final approval for publication. All authors read and approved the final manuscript.
